# Consumption patterns and factors associated with inappropriate prescribing of benzodiazepines in Primary Health Care settings

**DOI:** 10.1371/journal.pone.0309984

**Published:** 2024-09-04

**Authors:** Maria Olívia Barboza Zanetti, Iara dos Santos, Júlia Casanova Durante, Fabiana Rossi Varallo, Leonardo Régis Leira Pereira, Adriana Inocenti Miasso

**Affiliations:** 1 Department of Psychiatric Nursing and Human Sciences, University of São Paulo at Ribeirão Preto College of Nursing, Ribeirão Preto, São Paulo, Brazil; 2 Department of Pharmaceutical Sciences, University of São Paulo at School of Pharmaceutical Sciences of Ribeirao Preto, Ribeirão Preto, São Paulo, Brazil; Ekiti State University College of Medicine, NIGERIA

## Abstract

**Background:**

Benzodiazepines are frequently prescribed to treat anxiety and insomnia, but long-term use has been associated with the development of dependence, tolerance, and cognitive decline, especially among older adults. This study aimed to investigate the pattern of consumption and factors associated with inappropriate prescribing of benzodiazepines in primary health care.

**Methods:**

This is a cross-sectional analytical study, using dispensing records of diazepam, clonazepam, and nitrazepam from public pharmacies in a Brazilian municipality between 2018 and 2022. Metrics for benzodiazepine consumption were DDD (Defined Daily Dose) and DDD/1000PD (per 1000 population per day). Long-term/prolonged benzodiazepine use was defined as consuming at least 90 DDD and at least 2 dispensations per year. To ascertain associations between long-term use and predictor variables, a multivariate logistic regression model was utilized.

**Findings:**

A total of 40402 participants were included, with an average age of 55 years (SD = 0.30), 38.5% were older aged. Diazepam and nitrazepam exceeded the daily dose recommended. There was a reduction in diazepam consumption during the study period, as calculated by DDD/1.000PD, while the consumption of other benzodiazepines remained stable. However, a significant increase in diazepam consumption is noted when considering the last decade. Prolonged use was observed in 29.1% of participants, with a significant prevalence among the older people (34.8% of them were long-term users) and advancing age was identified as a risk factor for long-term use. Higher PDDs were also associated with long-term use and aging. Participants who used different benzodiazepines during the period had a higher risk of prolonged use.

**Conclusions:**

These results provide insights into the prevalence of problematic utilization of benzodiazepines in primary health care. Authorities and health care providers must take steps to encourage gradual cessation of prolonged benzodiazepine prescriptions and the embrace of suitable strategies for addressing anxiety and insomnia within primary health care settings.

## Introduction

According to the World Health Organization (WHO), Brazil has the highest prevalence of anxiety disorders globally, affecting 9.3% of its population [1]. A population survey conducted in 2023 reveals even more concerning data, indicating an anxiety prevalence of 26.8%, with 58.9% of Brazilians reporting a good perception of sleep [2]. Correspondingly, Brazil shows an increasing consumption of psychotropic drugs. Benzodiazepines (BZDs), commonly used anxiolytics for managing anxiety and insomnia, rank among the top five controlled medications sold in pharmacies [3]. Primary Health Care (PHC) serves as the primary source of BZD prescriptions in Brazil, which is in line with the fact that the main complaints related to mental health in PHC include anxiety, mood and sleep disorders [4].

While benzodiazepines are generally considered safe and effective when used in isolation and for short durations, prolonged usage is linked to the development of tolerance and dependence, casting doubt on its appropriateness [5]. Additionally, adverse effects stemming from benzodiazepine use encompass a decline in cognitive functions, memory, attention, and concentration, as well as mental confusion, delirium, changes in motor coordination, and disruptions in sleep patterns [6]. Combining benzodiazepines with certain medications, alcohol, or illicit substances can escalate risks, potentially inducing respiratory depression and leading to fatal outcomes [6]. These hazards underscore the necessity of monitoring usage duration and potential interactions for individuals prescribed benzodiazepines.

In older adults, the psychomotor side effects associated with benzodiazepines (BZDs) are significant contributors to falls, fractures, and hospital admissions [7]. These adverse effects also limit mobility and ultimately diminish an individual’s social engagement. Furthermore, geriatric syndromes predispose older adults to pharmacokinetic and pharmacodynamic alterations, potentially resulting in the accumulation of BZDs in the body. Additionally, the prevalence of polypharmacy among older adults increases their susceptibility to drug interactions. Due to these concerns, benzodiazepines are regarded as potentially inappropriate medications for older adults [7].

This research aimed to investigate consumption patterns and factors associated with the inappropriate prescribing of BZD in Primary Health Care settings.

## Methods

### Study design

This is an analytical, observational, cross-sectional study, conducted in PHC in the Municipality of Ribeirão Preto, SP, Brazil. Consumption and prevalence data of BZDs were estimated, and associations with sociodemographic factors were measured. To ensure methodological quality, this study adhered to the recommendations of the STROBE checklist (*STrengthening the Reporting of OBservational studies in Epidemiology*) for cross-sectional studies [8].

### Participants

All individuals with a record of dispensation of at least one BZD between the years 2018 and 2022 in any pharmacies within the PHC were considered eligible for inclusion in the study. Dispensation records with pharmaceutical forms for parenteral use (diazepam 5 mg/mL injectable solution) or with up to three units of BZD tablets were excluded, as this indicated episodic use for the management of acute anxiety situations within the healthcare institution.

To investigate the occurrence of drug interactions involving BZDs, a subset of the study population was considered by calculating a sample from individuals with a record of BZD dispensation. The exact number of PHC users in Ribeirão Preto receiving BZDs prescriptions is unknown. Therefore, the worst-case scenario was chosen for sample size calculation to maximize the sample size. In doing so, the prevalence of the investigated event was assumed to be 50%. A confidence level of 95% and an absolute precision of 1% for the proportion estimate were considered, resulting in a minimum sample size of 9.604 subjects randomly selected from all BZDs dispensation records.

### Variables and data collection

The information was collected from the computerized database of the Municipal Health Department of Ribeirão Preto, SP. The data were accessed for research purposes from March 2023 to July 2023. All participants had their dispensation records analyzed to extract the necessary data for estimating consumption, including the total quantity of BZDs dispensed in milligrams each year of the study and the number of days for which the BZD was prescribed each year. Additionally, demographic variables (gender, age at the date of the last dispensation, dispensation location) and clinical/pharmacological variables (quantity withdrawn and dose, BZD dispensed, and dispensation frequency) were extracted. The authors did not have access to information that could identify individual participants during or after data collection. The BZDs considered for analysis, listed in Ribeirão Preto’s Municipal List of Essential Medicines (REMUME) for home use, include diazepam (5 mg and 10 mg tablets), clonazepam (2 mg tablet and oral solution drops 2.5 mg/ml), and nitrazepam (5 mg tablet). Therefore, these were the three evaluated BZDs.

The consumption of BZD and the average annual BZD dispensed to each user were measured in milligrams per day, considering the years 2018 to 2022. The metrics used for the annual consumption estimate in the study were:

Defined Daily Dose per 1000 Inhabitants per day (DDD/1000PD) through the following calculation: DDD per 1000 PD = (annual milligrams of BZD dispensed X 1000 inhabitants) ÷ (DDD X 365 X estimated population for the year). Where: annual milligrams = total milligrams of BZD dispensed in the municipality each year analyzed; DDD = Defined Daily Dose established by the WHO.

- Defined Daily Dose per 1000 Inhabitants per day adjusted for the population that effectively uses the Brazilian Unified Health System (SUS), estimated at 75% (DDD75%/1000PD). Thus, the following calculation was performed: DDD ÷ 1000 PD = (annual milligrams of BZD dispensed X 1000 inhabitants) ÷ (DDD X 365 X 75% of the estimated population for the year).
- Prescribed Daily Dose (PDD): PDD = total quantity of BZD dispensed in milligrams per year divided by the number of days the patient used BZD in the year.

Prolonged use of BZD was defined as consumption of at least 90 DDD or more, equivalent to at least 3 months of use, and at least 2 separate dispensations of BZD during one year [9].

To evaluate the utilization of benzodiazepines (BZD) among older adults, individuals aged 60 years or older were considered, in accordance with the Brazilian Older Adult Statute and the World Health Organization’s definition for developing countries [10, 11].

For the subset of participants whose drug interactions were analyzed, data on other medications dispensed to the user were also extracted. Polypharmacy was defined as the use of five or more medications, which is the most widely accepted definition in the literature [12]. The Anatomical Therapeutic Chemical (ATC) classification system, proposed by the WHO, was employed to categorize dispensed medications based on their therapeutic classes, focusing on the first three levels of the system [13].

For the analysis of the frequency of drug interactions involving BZDs, the presence of other medications with potential for interaction in the dispensation records containing BZD was considered. Only medications prescribed during the period in which the participant used BZD were considered for analysis. Given the variability of medications in different databases, it was decided to evaluate interactions by consulting two sources of information (*Micromedex* and *Drugs*.*com*), increasing the accuracy of the observation. The BZD "nitrazepam" is not registered in the *Drugs*.*com* database; therefore, for interactions involving this medication, only *Micromedex* was consulted.

The medications dispensed for each participant, along with the BZD, were inputted into these platforms, and the positive cases for drug interactions were compiled based on severity levels: contraindicated (concomitant use of the medications is contraindicated); major (potentially fatal or requiring medical intervention); moderate severity (may result in exacerbation of the case or requires a change in pharmacotherapy); or minor (limited clinical effects). In case of discrepancies between the two information sources, the classification with the higher severity level was considered. Interactions were classified as pharmacokinetic or pharmacodynamic.

### Statistical analysis

The database was created in Microsoft Office Excel®, and statistical analysis was conducted using the SAS program version 9.4. An exploratory data analysis was performed, considering measures of central tendency and dispersion. The mean and median were calculated as measures of central tendency, and the standard deviation, interquartile range, as well as minimum and maximum values were calculated as measures of dispersion. Qualitative variables were summarized using absolute and relative frequencies.

To determine which of the predictor variables (gender, age, and medication) are associated with prolonged use of BZD, a multivariable logistic regression model was employed. This model estimates the Odds Ratio (OR) as the effect size measure. Multivariate linear regression models were constructed considering the outcomes of PDD for clonazepam, diazepam, and nitrazepam, with age, gender, and prolonged use of BZD as covariates in the model. To provide a better understanding of the relationship between age and PDD, in addition to linear age, squared age was included in the model as an independent variable to assess the quadratic effect of age on PDD.

The Pearson correlation coefficient was estimated to examine the relationship between the number of drug interactions and age, the number of prescribed medications, and the number of prescribed continuous-use medications.

The tests were conducted with a significance level of 5% and a confidence interval of 95%.

### Protection of study participants

The study protocol was approved by the Research Ethics Committee of University of São Paulo at Ribeirão Preto College of Nursing (CAAE: 64894522.8.0000.5393) and authorized by the Municipal Health Department of Ribeirão Preto. This study was conducted in accordance with the principles of the Declaration of Helsinki, ensuring the confidentiality of individual information and anonymity.

## Results

Between 2018 and 2022, a total of 40402 individuals received BZD from PHC pharmacies in Ribeirão Preto. The average age of these individuals at the date of the last recorded dispensation was 55 years (SD = 0.30). Among them, 938 individuals (2.3%) were under the age of 20, 23925 (59.2%) were aged between 20 and 59, and 15539 (38.5%) were aged 60 or older. Hence, it is evident that over one-third of the participants fall into the older age category. In terms of gender distribution, the majority of the population consisted of females, comprising 27360 individuals (67.7%). (Table 1). For comparison purposes, in 2022, the population of Ribeirão Preto consisted of 52.4% women and 47.6% men; 14% of the population was under 20 years old, 59.7% were aged between 20 and 59, and 17.4% were 60 years old or older. From a purely descriptive standpoint, the population with prolonged BZD use appears to be older and predominantly female [14].

**Table 1:** Variation in the consumption of benzodiazepines during the study period, according to the age and gender of users.

Year	Population	Individuals who obtained BZD	Age								Female Gender	
			0–19		20–59		≥ 60		Mean	SD		
			n	%	n	%	n	%			n	%
2018	694534	21275	237	1.1%	11019	51.8%	10019	47.1%	56	2	14940	70.2%
2019	703293	20900	253	1.2%	11086	53.0%	9560	45.7%	55	2	14746	70.6%
2020	711825	19244	250	1.3%	10262	53.3%	8731	45.4%	55	2.2	13554	70.4%
2021	720116	19065	251	1.3%	10496	55.1%	8318	43.6%	55	1.6	13416	70.4%
2022	698259	18382	227	1.2%	10194	55.5%	7959	43.3%	55	1.5	13028	70.9%
Total		40402	938	2.3%	23925	59.2%	15539	38.5%	55	0.3	27360	67.7%

BZD = benzodiazepines; SD = Standard Deviation; n = number.

When comparing the consumption of the three BZD available in the REMUME, diazepam and nitrazepam exhibited an average PDD surpassing the WHO’s recommended DDD in all study years (Table 2), implying a higher dosage consumption.

**Table 2:** Benzodiazepine consumption comparison, according to the study years.

Drug	WHO’s recommended DDD	Metric	Annual values					2018–2022 annual means	SD
			2018	2019	2020	2021	2022		
Clonazepam	8	PDD	2.4	2.5	2.6	2.5	2.5	2.5	0.07
		DDD/1000PD	3.2	3.1	3.2	3.1	3.1	3.1	0.05
		DDD75%/1000PD	4.6	4.5	4.6	4.4	4.4	4.5	0.10
Diazepam	10	PDD	11.0	11.8	12.1	11.9	12.1	11.9	0.45
		DDD/1000PD	5.5	5.6	5.2	4.8	4.7	5.2	0.40
		DDD75%/1000PD	7.8	8.0	7.4	6.9	6.7	7.4	0.56
Nitrazepam	5	PDD	6.6	6.4	6.7	6.7	7.2	6.7	0.29
		DDD/1000PD	0.2	0.2	0.2	0.2	0.2	0.2	0.00
		DDD75%/1000PD	0.3	0.3	0.2	0.2	0.2	0.2	0.05

PDD = Prescribed Daily Dose; DDD = Defined Daily Dose; PD = patients/day; SD = standard deviation.

Dispensation data presented in DDD/1000PD can provide an approximate estimate of the proportion of the study population treated daily with a specific medication or group of medications. For instance, 3.2 DDD/1000PD (clonazepam consumption in 2018) can be interpreted as follows: in a representative group of 1000 inhabitants, an average of 3.2 DDDs of clonazepam was utilized on any given day throughout the year 2018. Alternatively, this can be expressed as 3.2/1000 (0.32%) of the population received clonazepam every day in that year.

There are indications of a reduction in consumption, calculated by DDD/1000PD and DDD75%/1000PD, for diazepam, and maintenance in the consumption of other BZD over the years of the study (Table 2).

Despite the decreasing trend, diazepam remains the most widely consumed BZD in the municipality, particularly when considering the population that effectively utilizes the SUS (Table 2). Utilizing the rationale of DDD/1000PD, 0.74% of the SUS-utilizing population received diazepam daily in each year of the study, compared to 0.45% for clonazepam and 0.02% for nitrazepam.

Evidence was obtained indicating that higher PDD of clonazepam, diazepam, and nitrazepam are associated with prolonged use of these BZD. Being male is correlated with higher PDD for clonazepam and diazepam. Increasing age is associated with an increase in PDD for diazepam and clonazepam (Table 3).

**Table 3:** Estimates from the multivariate linear regression model, considering the outcome variable as the PDD of clonazepam, diazepam, and nitrazepam.

Drug	Variable	Parameter Estimate	Standard Error	T-Statistic	p-value
Clonazepam	Age	-0.0003	0.0003	-1.1300	0.2590
	Age-Squared	-0.0000	0.0000	-2.66	0.0077
	Gender	-0.0487	0.0120	-4.0600	< .0001
	Prolonged use	1.0208	0.0148	68.8000	< .0001
Diazepam	Age	-0.011	0.002	-5.050	< .0001
	Age-Squared	-0.0001	0.0000	-6.89	< .0001
	Gender	-0.677	0.077	-8.760	< .0001
	Prolonged use	2.691	0.076	35.490	< .0001
Nitrazepam	Age	-0.009	0.005	-1.830	0.068
	Age-Squared	-0.0001	0.0000	-1.81	0.0705
	Gender	0.058	0.200	0.290	0.773
	Prolonged use	0.690	0.183	3.770	0.001

It was observed that 29.1% of the participants engaged in prolonged use of BZDs. Among older individuals alone, 34.8% of them (n = 5412) were identified as long-term users. Nitrazepam and diazepam appear to be more frequently used for extended durations compared to clonazepam. Users who utilized different BZDs at various times over the five-year study period also demonstrated a higher frequency of prolonged use. Particularly noteworthy is the elevated prevalence of prolonged use among those who utilized all three available BZDs listed in the REMUME (92.2%), as well as among users of diazepam and nitrazepam (93.8%) (Table 4).

**Table 4:** Distribution of gender, drug, and age variables in relation to prolonged use of BZD.

	Prolonged use of BZD	
	No	Yes
	n (%)	n (%)
**Gender**		
Female	19438 (71.1)	7922 (29.0)
Male	9197 (70.5)	3844 (29.5)
**Drug**		
Clonazepam	22538 (84.4)	4161 (15.6)
Clonazepam and Diazepam	1540 (37.9)	2524 (62.1)
Clonazepam and Nitrazepam	36 (24.8)	109 (75.2)
Clonazepam, Diazepam and Nitrazepam	7 (7.8)	83 (92.2)
Diazepam	4454 (48.4)	4755 (51.6)
Diazepam and Nitrazepam	3 (6.3)	45 (93.8)
Nitrazepam	57 (39.0)	89 (61.0)
**Age** *	51.57 (18.5)	57.15 (15.2)

*Mean and standard deviation values. The variables gender and drug were summarized considering absolute and relative frequencies.

It can be inferred that the gender variable is not a predictor for prolonged use of BZDs. However, for each one-year increase in the age of the users, there is 1.022 times increase in the risk of prolonged use of BZDs. In terms of the specific BZD used, individuals who exclusively used clonazepam exhibited the lowest risk of prolonged use. Those who exclusively used diazepam had a lower risk of prolonged use compared to those who used nitrazepam. Moreover, individuals who used different BZDs at different times over the five years generally had a higher risk of prolonged BZD use compared to those who consistently used only one BZD throughout the period (whether it be clonazepam, diazepam, or nitrazepam) (Table 5).

**Table 5:** Estimate of the adjusted odds ratio by the logistic regression model considering prolonged use of BZD as the outcome.

	Odds Ratio	CI 95%	
		LL	UL
Female vs Male	1.03	0.98	1.08
Age	1.02	1.02	1.02
vs Nitrazepam			
Clonazepam	0.11	0.08	0.15
Diazepam	0.65	0.46	0.91
Clonazepam and diazepam	1.01	0.71	1.43
Clonazepam and nitrazepam	1.95	1.17	3.26
Diazepam and nitrazepam	8.64	2.55	29.26
Clonazepam, diazepam and nitrazepam	7.86	3.37	18.32
vs Clonazepam			
Diazepam	6.08	5.76	6.41
Nitrazepam	9.42	6.67	13.29
Clonazepam and diazepam	9.49	8.82	10.21
Clonazepam and nitrazepam	18.40	12.54	27.00
Diazepam and nitrazepam	81.41	25.24	262.55
Clonazepam, diazepam and nitrazepam	73.98	34.07	160.62
vs Diazepam			
Clonazepam	0.17	0.16	0.17
Nitrazepam	1.55	1.10	2.19
Clonazepam and diazepam	1.56	1.45	1.69
Clonazepam and nitrazepam	3.03	2.06	4.45
Diazepam and nitrazepam	13.40	4.15	43.21
Clonazepam, diazepam and nitrazepam	12.17	5.61	26.44

LL = Lower limit; UL = Upper limit; CI: Confidence interval.

Despite diazepam being the most extensively consumed benzodiazepine (BZD) in the municipality based on consumption parameters, a higher number of individuals used clonazepam. Specifically, clonazepam was utilized by 76.7% (n = 30999) of individuals who obtained BZDs over the five years of the study. From this observation, it can be concluded that although a greater number of inhabitants use clonazepam, its utilization is at lower doses, durations, or frequencies compared to inhabitants who use diazepam.

For the assessment of drug interactions, 13662 individuals with records of BZD dispensation in public pharmacies of Ribeirão Preto were considered. Focusing solely on continuous-use medications, the average number of prescribed medications per BZD user was 5.12 (SD = 3.59), ranging from one to 27 medications. A total of 6531 (47.8%) BZD users were in polypharmacy.

A total of 51190 interactions involving BZD were identified, with an average of 3.75 interactions per user (SD = 4.07), ranging from none to 37 interactions for a single user. It was observed that 11331 (82.9%) users presented at least one drug interaction involving BZD. Among the identified interactions, 15951 were classified as pharmacokinetic, 35186 as pharmacodynamic, and 53 had an unknown mechanism. The drug interactions are described in S1 Table. Concerning the severity of interactions involving BZD, 861 (1.7%) were classified as major, 45733 (89.3%) as moderate, and 4596 (9.0%) as minor. No interaction was considered a contraindication. The most frequent interactions involved antidepressant drugs (30.0%), antipsychotics (11.5%), and medications for peptic ulcer and gastroesophageal reflux disease (10.5%). S2 Table provides a detailed presentation of the dispensed medications that interact with BZD, grouped according to their therapeutic classes.

The Pearson coefficient estimate indicates a relationship between the number of drug interactions obtained and the increase in age (p < 0.0001) as well as the increase in the number of prescribed continuous-use medications (p < 0.0001).

## Discussion

Brazil ranks as the third largest consumer of BZDs globally [15], a trend evident in the presented findings. A comparison with a study on psychotropic drug consumption conducted in Ribeirão Preto/SP in 2012 reveals slight increases in the PDD for clonazepam (2012: 2.45; 2022: 2.5), substantial increases in the PDD for diazepam (2012: 6.43; 2022: 12.1), and nitrazepam (2012: 8.4; 2022: 7.2) remains above the recommended DDD despite its reduced consumption [16]. Furthermore, 29.1% of the study population is engaged in prolonged use of BZD, which may lead to a clinically significant condition of physical and psychological dependence [17].

Discrepancies between the WHO’s DDD and the PDD may arise; however, when significant, they can be used as a strategy for screening users eligible to initiate a deprescription process of BZD, aiming to contribute to the safe use of these medications.

The PDDs obtained for diazepam and nitrazepam surpass the WHO’s recommended DDDs. Correspondingly, diazepam, nitrazepam, and combinations of BZDs were more frequently utilized over the long term compared to clonazepam. The study suggests that higher PDDs are linked to prolonged BZD use, indicating that as BZDs are employed for longer durations, their doses tend to be higher. These findings raise concerns regarding acquired tolerance associated with prolonged BZD use. Diazepam and nitrazepam, being more frequently used for extended periods, may see increased doses due to tolerance development. Moreover, the association between the use of multiple BZDs over the study period and prolonged use also suggests that acquired tolerance from prolonged use may facilitate the substitution of BZDs.

Despite being widely used by the study population, clonazepam showed a lower risk of prolonged use. This may be related to its use in lower doses, resulting in less induction of tolerance and dependence. The literature indicates that higher doses of BZD are associated with a faster induction of dependence and that prolonged use, even at therapeutic doses, is also linked to dependence [17]. High-potency benzodiazepines with short elimination half-lives can induce dependence more rapidly than those with lower potency and longer elimination half-lives [17, 18]. Clonazepam is a high-potency benzodiazepine with an intermediate elimination half-life, while diazepam has lower potency and a long elimination half-life [19]. Based on its pharmacokinetic characteristics, clonazepam is a benzodiazepine with the potential to induce dependence more rapidly [18, 19]. This may justify greater caution in its prescription, such as prescribing lower doses and for shorter periods of time.

Nitrazepam is a BZD standardized in the municipality of Ribeirão Preto as a hypnotic and sedative, but not as an anxiolytic, due to its lower potency and elimination half-life [19]. Its therapeutic indication may justify the lower prescription frequency, especially considering that clonazepam and diazepam are more commonly prescribed and well-known BZDs in Brazil.

The high consumption of BZD by women and older adults is supported by other studies [5, 16, 20]. The difference between genders in medication use may be related to the fact that traditionally, women are more concerned with self-care and tend to use healthcare services more frequently [20]. Additionally, the construction of the Brazilian healthcare system has historically focused on maternal-child health, bringing women closer to such services [20]. It should be noted that anxiety disorders are more prevalent in women [21, 22], possibly influenced by fatigue and the accumulation of responsibilities imposed by a social role as caregivers and family managers, in addition to the need to participate in the workforce [20, 21].

Despite higher BZD consumption in women, gender was not considered a predictor for prolonged use. Being male is associated with higher PDD of clonazepam and diazepam. A systematic literature review investigating inappropriate BZD use yielded conflicting results regarding gender, suggesting that the influence of gender on inappropriate use is likely modest and may be affected by sample size, covariates (user age, concurrent drug use, prescribed dosage), and methodological variations among studies [23].

Older adults constitute a substantial portion of the BZD users in the study (38.5%), which is alarming. Furthermore, evidence indicates that with increasing age, there is an increase in prolonged BZD use, and older adults use higher PDD of diazepam and clonazepam. The sedative properties of BZD are attractive for use by older adults, as intrinsic physiological changes associated with aging alter sleep patterns [18]. However, BZDs are considered potentially inappropriate medications for older adults, as this age group generally exhibits increased sensitivity to BZD, decreased metabolism of long-acting agents (such as diazepam), and uses medications for managing other health conditions with potential for drug interactions [7, 18]. All BZD increase the risk of cognitive impairment, delirium, falls, fractures, and automobile accidents in older adults [7, 20].

The results indicate a decrease in diazepam consumption between 2018 and 2022, which may be related to the COVID-19 pandemic. Despite a 25% increase in symptoms of anxiety and depression [24], the need for social isolation has brought about changes in the flow of healthcare services. In Brazil, mental health services were provided by 80% of PHC Units, with 68% maintaining them and 12% adapting to telehealth tactics [25]. These circumstances have hindered access to mental health care, as well as access to psychotropic medications [25].

Despite the high average number of drug interactions involving BZD per participant (3.75; SD = 4.07), most interactions were considered to have moderate severity. An example is the most frequently encountered interaction, clonazepam with sertraline, which is a quite common combination recommended in clinical protocols and therapeutic guidelines [18]. This combination is beneficial in the initial treatment of an individual with anxiety or depression [18]. However, several interactions were classified by information sources as moderate, even though it is known that these combinations require strict monitoring.

In this study, an association was found between BZDs and barbiturates (phenobarbital), tricyclic antidepressants (amitriptyline, nortriptyline, imipramine), opioids (tramadol), antihistamines (dimenhydrinate, promethazine), and dopamine receptor antagonists (biperiden). Although only tramadol was considered important by information sources, all are examples of pharmacodynamic interactions involving BZD [19]. The addition of these central nervous system (CNS) depressant drugs to BZD can be dangerous, as it has the potential to cause respiratory depression and death [26]. Even if considered moderate, such interactions necessitate monitoring for excessive or prolonged CNS and respiratory depression [19, 26]. Outpatients should be warned about the possibility of additive effects on the CNS (drowsiness, dizziness, fainting, or confusion) and advised to avoid activities requiring alertness, especially at the beginning of use [18, 19].

The interaction between BZDs and tramadol is considered important and occurred in 340 individuals in the study. In the United States, emergency department admissions due to combinations containing BZDs and opioids increased by more than 300% between 2004 and 2011 [27, 28]; BZD were involved in almost 30% of opioid overdose deaths in this country in 2015 [23]. Although no data of hospital admissions or overdoses in Brazil due to the associated use of opioids and BZDs were found in the literature, the use of opioids is increasing significantly in the country, and the association with BZDs is concerning [29, 30].

Pharmacokinetic interactions involving BZDs often stem from metabolic changes, as BZDs are primarily metabolized in the liver by enzymes of the CYP450 family [18, 19]. Concurrent administration of BZDs with drugs that inhibit these hepatic enzymes can elevate serum concentrations of BZDs, leading to intensified adverse effects [18, 26]. Examples identified in this study include interactions with antidepressants (fluoxetine), proton pump inhibitors (omeprazole), azole antifungals (fluconazole), macrolide antibiotics (clarithromycin, erythromycin), and antiretrovirals (darunavir, efavirenz, nevirapine, ritonavir, tenofovir + lamivudine + efavirenz, zidovudine + lamivudine). While these interactions were categorized as moderately relevant, vigilance for excessive or prolonged sedation is crucial, and alternative therapeutic options that do not inhibit these enzymes should be prioritized [19, 26].

Drugs capable of inhibiting the synthesis of CYP isoenzymes, such as in the case of combinations containing rifampicin found in this study, may decrease serum levels of BZD and reduce their therapeutic effect [26]. Although this interaction is considered secondary as it does not pose a safety concern, the lack of therapeutic response to BZD can be detrimental to the user, and its effect should be monitored [19].

It should also be noted that the number of drug interactions increases with age (p<0.0001) and with the increase in the number of prescribed continuous-use medications (p<0.0001). Therefore, exposure to drug interactions further raises the risk of adverse events involving BZD in older adults.

One limitation of this study is the potential overestimation of consumption data, inherent to the retrospective analysis of dispensing records, as the dispensing of medication does not necessarily indicate actual consumption. Additionally, potential medication shortages that may have occurred during the analyzed period, preventing dispensing, could not be evaluated because information on stockouts was not recorded in the computerized system. Moreover, the system’s data may contain errors in user registration or dispensing records since they are recorded by different professionals. While it is important to acknowledge the limitation of not having access to certain clinical information, such as specific diagnoses, it is worth noting that the prevalence of diagnoses warranting BZD use in PHC settings prominently includes anxiety and depression [4, 31]. Additionally, BZD use is inappropriate among older adults regardless of the clinical indication [7, 32]. Therefore, while our study does not permit generalized conclusions, it sheds light on concerning patterns of BZD consumption that align with established clinical practices and guidelines.

Despite the limitations, this study presents several strengths that contribute to its robustness and relevance. One of the main strengths lies in the adopted analysis method, which employed a comprehensive approach to assess BZD consumption and potential drug interactions in a representative sample of the population. By considering not only the frequency of prescriptions but also the dispensed doses and usage patterns over time, this study provides a detailed and dynamic insight into BZD use in PHC, highlighting the main issues related to inappropriate use. Furthermore, the inclusion of a wide range of demographic and clinical variables allows for a more comprehensive analysis of the factors associated with the consumption of these medications and their interactions with other drugs. These methodological strengths, coupled with careful interpretation of the results, enhance the validity and relevance of the findings of this study, particularly to provide relevant information for the development of public policies aimed at the rational use of BZDs.

To prevent inappropriate use and prescription of BZD, efforts should focus on preventing prolonged use, motivating users to initiate gradual discontinuation of the medication, encouraging psychotherapy, integrative and complementary practices, and discussing with the healthcare team the risks of excessive medication in mood and sleep disorders [32]. It is necessary for PHC teams to operationalize their attributes of comprehensiveness and care coordination, serving as a space for guidance, awareness, and action to discuss the unique aspects associated with the demands of users of BZD monitored in APS, and, when necessary, in collaboration with mental health specialists [31].

Governments, administrators, and healthcare professionals need to implement validated evidence-based protocols or guidelines to promote the deprescription of BZD, as done by the College of Family Physicians of Canada [6]. The adoption of more appropriate approaches for managing anxiety and insomnia in PHC is also fundamental, such as psychological treatments provided by a specialist (psychologist or clinical psychologist) or non-specialists (GP, nurse, trainee), exercise, relaxation and sleep hygiene techniques [33–35]. Alternative and complementary approaches, such as light therapy, aromatherapy, music therapy, and herbal medicine, may also be relevant [35]. It is essential to individualize the management of anxiety and sleep disorders for the provision of person-centered care, taking into account the best scientific evidence, as well as clinical specificities, preferences, values, comfort, and human dignity.

## Conclusion

The findings of this study provide insights into the prevalence of problematic prescription and inappropriate utilization of BZDs in PHC settings. Despite not having access to patients’ clinical diagnoses, the analysis of dispensing and consumption data revealed concerning patterns, highlighting the prolonged use by 29.1% of individuals who obtained BZD from pharmacies in the municipality and significant use among older adults (38.5% of users). Advancing age was identified as a risk factor for prolonged BZD use and in higher PDD. Additionally, the study identified the presence of relevant drug interactions, underscoring the importance of vigilance in prescribing practices. Furthermore, it was observed a significant increase in diazepam consumption over the last decade. The limitations of data precludes generalized conclusions, nevertheless, these findings shed light on concerning trends in BZD consumption that warrant attention from healthcare providers and policymakers.

## Supporting information

S1 Table Description of the interactions involving BZD identified. (DOCX)

S2 Table Detailed presentation of the dispensed drugs that interact with BZD, grouped according to their therapeutic classes (ATC). (DOCX)
