# Supplementary material for: Consumption patterns and factors associated with inappropriate prescribing of benzodiazepines in Primary Health Care settings
Source: PLoS One. 2024 Sep 4;19(9):e0309984. doi: 10.1371/journal.pone.0309984 (PMC11373788; doi:10.1371/journal.pone.0309984)
Supplement: S1 Table — (DOCX) [file pone.0309984.s001.docx]

S1 Table. Description of the interactions involving BZD identified

| **Interactions** | **Severity** | **Quality of Information** | **Classification** | **Frequency** | **%** |
| --- | --- | --- | --- | --- | --- |
| CLONAZEPAM + VALPROIC ACID | MODERATE | NOT AVAILABLE | PHARMACOKINETICS | 768 | 1.50 |
| CLONAZEPAM + AMINOPHYLLINE | MODERATE | GOOD | PHARMACODYNAMICS | 22 | 0.04 |
| CLONAZEPAM + AMIODARONE | MODERATE | GOOD | PHARMACODYNAMICS | 134 | 0.26 |
| CLONAZEPAM + AMITRIPTYLINE | MODERATE | NOT AVAILABLE | PHARMACODYNAMICS | 1649 | 3.22 |
| CLONAZEPAM + AMLODIPINE | MODERATE | NOT AVAILABLE | PHARMACODYNAMICS | 1289 | 2.52 |
| CLONAZEPAM + ATENOLOL | MODERATE | NOT AVAILABLE | PHARMACODYNAMICS | 287 | 0.56 |
| CLONAZEPAM + BACLOFEN | MODERATE | REGULAR | PHARMACODYNAMICS | 19 | 0.04 |
| CLONAZEPAM + BIPERIDONE | MODERATE | NOT AVAILABLE | PHARMACODYNAMICS | 419 | 0.82 |
| CLONAZEPAM + BROMOPRIDE | MODERATE | REGULAR | PHARMACODYNAMICS | 52 | 0.10 |
| CLONAZEPAM + BUPROPION | MODERATE | NOT AVAILABLE | PHARMACODYNAMICS | 1 | 0.00 |
| CLONAZEPAM + CAPTOPRIL | MODERATE | NOT AVAILABLE | PHARMACODYNAMICS | 94 | 0.18 |
| CLONAZEPAM + CARBAMAZEPINE | MODERATE | GOOD | PHARMACOKINETICS | 590 | 1.15 |
| CLONAZEPAM + CALCIUM CARBONATE | SECUNDARY | NOT AVAILABLE | PHARMACOKINETICS | 385 | 0.75 |
| CLONAZEPAM + LITHIUM CARBONATE | MODERATE | NOT AVAILABLE | PHARMACODYNAMICS | 301 | 0.59 |
| CLONAZEPAM + CARVEDILOL | MODERATE | NOT AVAILABLE | PHARMACODYNAMICS | 536 | 1.05 |
| CLONAZEPAM + CLARITHROMYCIN | MODERATE | REGULAR | PHARMACOKINETICS | 2 | 0.00 |
| CLONAZEPAM + CLOMIPRAMINE | MODERATE | NOT AVAILABLE | PHARMACODYNAMICS | 386 | 0.75 |
| CLONAZEPAM + CLONIDINE | MODERATE | NOT AVAILABLE | PHARMACODYNAMICS | 387 | 0.76 |
| CLONAZEPAM + CHLORPROMAZINE | MODERATE | REGULAR | PHARMACODYNAMICS | 850 | 1.66 |
| CLONAZEPAM + DARUNAVIR | MODERATE | NOT AVAILABLE | PHARMACOKINETICS | 18 | 0.04 |
| CLONAZEPAM + DIGOXIN | MODERATE | NOT AVAILABLE | PHARMACOKINETICS | 44 | 0.09 |
| CLONAZEPAM + DIMENHYDRINATE + PYRIDOXINE (Inj. Sol.) | MODERATE | NOT AVAILABLE | PHARMACODYNAMICS | 3 | 0.01 |
| CLONAZEPAM + ISOSORBIDE DINITRATE | MODERATE | NOT AVAILABLE | PHARMACODYNAMICS | 1 | 0.00 |
| CLONAZEPAM + DOXAZOSIN | MODERATE | NOT AVAILABLE | PHARMACODYNAMICS | 134 | 0.26 |
| CLONAZEPAM + EFAVIRENZ | MODERATE | NOT AVAILABLE | PHARMACOKINETICS | 3 | 0.01 |
| CLONAZEPAM + ENALAPRIL | MODERATE | NOT AVAILABLE | PHARMACODYNAMICS | 1207 | 2.36 |
| CLONAZEPAM + ESMOLOL (Inj. Sol.) | MODERATE | NOT AVAILABLE | PHARMACODYNAMICS | 1 | 0.00 |
| CLONAZEPAM + SPIRONOLACTONE | MODERATE | NOT AVAILABLE | PHARMACODYNAMICS | 353 | 0.69 |
| CLONAZEPAM + ETHINYLESTRADIOL + LEVONORGESTREL | SECUNDARY | GOOD | PHARMACOKINETICS | 38 | 0.07 |
| CLONAZEPAM + PHENYTOIN | MODERATE | GOOD | PHARMACOKINETICS | 142 | 0.28 |
| CLONAZEPAM + PHENOBARBITAL | MODERATE | GOOD | PHARMACODYNAMICS | 128 | 0.25 |
| CLONAZEPAM + FLUCONAZOLE | MODERATE | REGULAR | PHARMACOKINETICS | 210 | 0.41 |
| CLONAZEPAM + FLUMAZENIL (Inj. Sol.) | MODERATE | REGULAR | PHARMACODYNAMICS | 1 | 0.00 |
| CLONAZEPAM + FLUOXETINE | MODERATE | NOT AVAILABLE | PHARMACODYNAMICS | 2283 | 4.46 |
| CLONAZEPAM + FUROSEMIDE | MODERATE | NOT AVAILABLE | PHARMACODYNAMICS | 670 | 1.31 |
| CLONAZEPAM + GABAPENTIN | IMPORTANT | REGULAR | PHARMACODYNAMICS | 1 | 0.00 |
| CLONAZEPAM + HALOPERIDOL | MODERATE | REGULAR | PHARMACODYNAMICS | 839 | 1.64 |
| CLONAZEPAM + HYDROCHLOROTHIAZIDE | MODERATE | NOT AVAILABLE | PHARMACODYNAMICS | 1855 | 3.62 |
| CLONAZEPAM +ALUMINUM HYDROXIDE | SECUNDARY | NOT AVAILABLE | PHARMACOKINETICS | 188 | 0.37 |
| CLONAZEPAM + IMIPRAMINE | MODERATE | NOT AVAILABLE | PHARMACODYNAMICS | 303 | 0.59 |
| CLONAZEPAM + ISONIAZID | MODERATE | NOT AVAILABLE | PHARMACOKINETICS | 5 | 0.01 |
| CLONAZEPAM + ISONIAZID + RIFAMPIN | MODERATE | NOT AVAILABLE | PHARMACOKINETICS | 1 | 0.00 |
| CLONAZEPAM + ISONIAZID + RIFAMPIN + PYRAZINAMIDE + ETHAMBUTOL | MODERATE | NOT AVAILABLE | PHARMACOKINETICS | 2 | 0.00 |
| CLONAZEPAM + LEVODOPA + CARBIDOPA | MODERATE | NOT AVAILABLE | UNKNOWN | 38 | 0.07 |
| CLONAZEPAM + LEVOMEPROMAZINE | IMPORTANT | REGULAR | PHARMACODYNAMICS | 402 | 0.79 |
| CLONAZEPAM + LOSARTAN | MODERATE | NOT AVAILABLE | PHARMACODYNAMICS | 2096 | 4.09 |
| CLONAZEPAM + METHYLDOPA | MODERATE | NOT AVAILABLE | PHARMACODYNAMICS | 29 | 0.06 |
| CLONAZEPAM + METOCLOPRAMIDE | MODERATE | REGULAR | PHARMACODYNAMICS | 74 | 0.14 |
| CLONAZEPAM + METOPROLOL (Inj. Sol.) | MODERATE | NOT AVAILABLE | PHARMACODYNAMICS | 5 | 0.01 |
| CLONAZEPAM + MIDAZOLAM (Inj. Sol.) | MODERATE | REGULAR | PHARMACODYNAMICS | 84 | 0.16 |
| CLONAZEPAM + ISOSORBIDE MONONITRATE | MODERATE | NOT AVAILABLE | PHARMACODYNAMICS | 122 | 0.24 |
| CLONAZEPAM + NEVIRAPINE | MODERATE | GOOD | PHARMACOKINETICS | 2 | 0.00 |
| CLONAZEPAM + NORTRIPTYLINE | MODERATE | NOT AVAILABLE | PHARMACODYNAMICS | 478 | 0.93 |
| CLONAZEPAM + OMEPRAZOLE | MODERATE | NOT AVAILABLE | PHARMACOKINETICS | 3251 | 6.35 |
| CLONAZEPAM + PERPHENAZINE | IMPORTANT | REGULAR | PHARMACODYNAMICS | 65 | 0.13 |
| CLONAZEPAM + PROMETHAZINE | MODERATE | REGULAR | PHARMACODYNAMICS | 747 | 1.46 |
| CLONAZEPAM + PROPRANOLOL | MODERATE | NOT AVAILABLE | PHARMACODYNAMICS | 591 | 1.15 |
| CLONAZEPAM + QUETIAPINE | MODERATE | REGULAR | PHARMACODYNAMICS | 1 | 0.00 |
| CLONAZEPAM + RISPERIDONE | MODERATE | NOT AVAILABLE | PHARMACODYNAMICS | 832 | 1.63 |
| CLONAZEPAM + RITONAVIR | MODERATE | GOOD | PHARMACOKINETICS | 39 | 0.08 |
| CLONAZEPAM + SERTRALINE | MODERATE | NOT AVAILABLE | PHARMACODYNAMICS | 4285 | 8.37 |
| CLONAZEPAM + TENOFOVIR + LAMIVUDINE + EFAVIRENZ | MODERATE | NOT AVAILABLE | PHARMACOKINETICS | 15 | 0.03 |
| CLONAZEPAM + THIORIDAZINE | MODERATE | REGULAR | PHARMACODYNAMICS | 62 | 0.12 |
| CLONAZEPAM + TRAMADOL | IMPORTANT | REGULAR | PHARMACODYNAMICS | 167 | 0.33 |
| CLONAZEPAM + VECURONIUM | MODERATE | NOT AVAILABLE | PHARMACODYNAMICS | 1 | 0.00 |
| CLONAZEPAM + ZIDOVUDINE + LAMIVUDINE | SECUNDARY | NOT AVAILABLE | PHARMACOKINETICS | 11 | 0.02 |
| DIAZEPAM + VALPROIC ACID | MODERATE | NOT AVAILABLE | PHARMACOKINETICS | 611 | 1.19 |
| DIAZEPAM + AMINOPHYLLINE | MODERATE | GOOD | PHARMACODYNAMICS | 25 | 0.05 |
| DIAZEPAM + AMIODARONE | SECUNDARY | NOT AVAILABLE | PHARMACODYNAMICS | 93 | 0.18 |
| DIAZEPAM + AMITRIPTYLINE | MODERATE | NOT AVAILABLE | PHARMACODYNAMICS | 1111 | 2.17 |
| DIAZEPAM + AMLODIPINE | MODERATE | NOT AVAILABLE | PHARMACODYNAMICS | 911 | 1.78 |
| DIAZEPAM + ATENOLOL | MODERATE | NOT AVAILABLE | PHARMACODYNAMICS | 203 | 0.40 |
| DIAZEPAM + BACLOFEN | MODERATE | NOT AVAILABLE | PHARMACODYNAMICS | 16 | 0.03 |
| DIAZEPAM + BIPERIDONE | MODERATE | NOT AVAILABLE | PHARMACODYNAMICS | 391 | 0.76 |
| DIAZEPAM + BROMOPRIDE | MODERATE | REGULAR | PHARMACODYNAMICS | 32 | 0.06 |
| DIAZEPAM + BUPROPION | MODERATE | NOT AVAILABLE | PHARMACODYNAMICS | 2 | 0.00 |
| DIAZEPAM + CAPTOPRIL | MODERATE | NOT AVAILABLE | PHARMACODYNAMICS | 79 | 0.15 |
| DIAZEPAM + CARBAMAZEPINE | MODERATE | NOT AVAILABLE | PHARMACOKINETICS | 517 | 1.01 |
| DIAZEPAM + CALCIUM CARBONATE | SECUNDARY | NOT AVAILABLE | PHARMACOKINETICS | 234 | 0.46 |
| DIAZEPAM + LITHIUM CARBONATE | SECUNDARY | NOT AVAILABLE | PHARMACODYNAMICS | 228 | 0.45 |
| DIAZEPAM + CARVEDILOL | MODERATE | NOT AVAILABLE | PHARMACODYNAMICS | 369 | 0.72 |
| DIAZEPAM + CIPROFLOXACIN | SECUNDARY | NOT AVAILABLE | PHARMACOKINETICS | 100 | 0.20 |
| DIAZEPAM + CLARITHROMYCIN | MODERATE | GOOD | PHARMACOKINETICS | 2 | 0.00 |
| DIAZEPAM + CLOMIPRAMINE | MODERATE | NOT AVAILABLE | PHARMACODYNAMICS | 244 | 0.48 |
| DIAZEPAM + CLONIDINE | MODERATE | NOT AVAILABLE | PHARMACODYNAMICS | 310 | 0.61 |
| DIAZEPAM + CHLORPROMAZINE | MODERATE | NOT AVAILABLE | PHARMACODYNAMICS | 677 | 1.32 |
| DIAZEPAM + DARUNAVIR | MODERATE | NOT AVAILABLE | PHARMACOKINETICS | 15 | 0.03 |
| DIAZEPAM + DEXAMETHASONE (Inj. Sol.) | SECUNDARY | NOT AVAILABLE | PHARMACOKINETICS | 3 | 0.01 |
| DIAZEPAM + DIGOXIN | MODERATE | REGULAR | PHARMACOKINETICS | 26 | 0.05 |
| DIAZEPAM + DIMENHYDRINATE + PYRIDOXINE (Inj. Sol.) | MODERATE | NOT AVAILABLE | PHARMACODYNAMICS | 3 | 0.01 |
| DIAZEPAM + ISOSORBIDE DINITRATE | MODERATE | NOT AVAILABLE | PHARMACODYNAMICS | 3 | 0.01 |
| DIAZEPAM + DOXAZOSIN | MODERATE | NOT AVAILABLE | PHARMACODYNAMICS | 88 | 0.17 |
| DIAZEPAM + EFAVIRENZ | MODERATE | NOT AVAILABLE | PHARMACOKINETICS | 7 | 0.01 |
| DIAZEPAM + ENALAPRIL | MODERATE | NOT AVAILABLE | PHARMACODYNAMICS | 816 | 1.59 |
| DIAZEPAM + ERITHROMYCIN | MODERATE | GOOD | PHARMACOKINETICS | 1 | 0.00 |
| DIAZEPAM + ESMOLOL (Inj. Sol.) | MODERATE | NOT AVAILABLE | PHARMACODYNAMICS | 1 | 0.00 |
| DIAZEPAM + SPIRONOLACTONE | MODERATE | NOT AVAILABLE | PHARMACODYNAMICS | 254 | 0.50 |
| DIAZEPAM + ESTRADIOL | MODERATE | GOOD | PHARMACOKINETICS | 7 | 0.01 |
| DIAZEPAM + ESTRADIOL + NORETHISTERONE | MODERATE | GOOD | PHARMACOKINETICS | 45 | 0.09 |
| DIAZEPAM + ETHINYLESTRADIOL + LEVONORGESTREL | SECUNDARY | GOOD | PHARMACOKINETICS | 24 | 0.05 |
| DIAZEPAM + PHENYTOIN | MODERATE | GOOD | PHARMACOKINETICS | 156 | 0.30 |
| DIAZEPAM + PHENOBARBITAL | MODERATE | GOOD | PHARMACODYNAMICS | 152 | 0.30 |
| DIAZEPAM + FLUCONAZOLE | MODERATE | NOT AVAILABLE | PHARMACOKINETICS | 127 | 0.25 |
| DIAZEPAM + FLUMAZENIL (Inj. Sol.) | MODERATE | NOT AVAILABLE | PHARMACODYNAMICS | 1 | 0.00 |
| DIAZEPAM + FLUOXETINE | MODERATE | GOOD | PHARMACOKINETICS | 1511 | 2.95 |
| DIAZEPAM + FUROSEMIDE | MODERATE | NOT AVAILABLE | PHARMACODYNAMICS | 455 | 0.89 |
| DIAZEPAM + HALOPERIDOL | MODERATE | NOT AVAILABLE | PHARMACODYNAMICS | 854 | 1.67 |
| DIAZEPAM + HYDRALAZINE (Inj. Sol.) | MODERATE | NOT AVAILABLE | PHARMACODYNAMICS | 2 | 0.00 |
| DIAZEPAM + HYDROCHLOROTHIAZIDE | MODERATE | NOT AVAILABLE | PHARMACODYNAMICS | 1239 | 2.42 |
| DIAZEPAM + ALUMINUM HYDROXIDE | SECUNDARY | NOT AVAILABLE | PHARMACOKINETICS | 123 | 0.24 |
| DIAZEPAM + IMIPRAMINE | MODERATE | NOT AVAILABLE | PHARMACODYNAMICS | 207 | 0.40 |
| DIAZEPAM + ISONIAZIDE | MODERATE | GOOD | PHARMACOKINETICS | 3 | 0.01 |
| DIAZEPAM + ISONIAZIDE + RIFAMPIN | MODERATE | GOOD | PHARMACOKINETICS | 1 | 0.00 |
| DIAZEPAM + ISONIAZID + RIFAMPIN + PYRAZINAMIDE + ETHAMBUTOL | MODERATE | GOOD | PHARMACOKINETICS | 3 | 0.01 |
| DIAZEPAM + LEVODOPA + CARBIDOPA | MODERATE | NOT AVAILABLE | UNKNOWN | 15 | 0.03 |
| DIAZEPAM + LOSARTAN | MODERATE | NOT AVAILABLE | PHARMACOKINETICS | 1367 | 2.67 |
| DIAZEPAM + METHYLDOPA | MODERATE | NOT AVAILABLE | PHARMACODYNAMICS | 29 | 0.06 |
| DIAZEPAM + METOCLOPRAMIDE | MODERATE | REGULAR | PHARMACODYNAMICS | 58 | 0.11 |
| DIAZEPAM + METOPROLOL (Inj. Sol.) | SECUNDARY | NOT AVAILABLE | PHARMACOKINETICS | 6 | 0.01 |
| DIAZEPAM + MIDAZOLAM (Inj. Sol.) | MODERATE | REGULAR | PHARMACODYNAMICS | 102 | 0.20 |
| DIAZEPAM + ISOSORBIDE MONONITRATE | MODERATE | NOT AVAILABLE | PHARMACODYNAMICS | 87 | 0.17 |
| DIAZEPAM + NORETHISTERONE | MODERATE | GOOD | PHARMACOKINETICS | 2 | 0.00 |
| DIAZEPAM + NORTRIPTYLINE | MODERATE | NOT AVAILABLE | PHARMACODYNAMICS | 327 | 0.64 |
| DIAZEPAM + OMEPRAZOLE | MODERATE | GOOD | PHARMACOKINETICS | 2139 | 4.18 |
| DIAZEPAM + PERICIAZINE | IMPORTANT | REGULAR | PHARMACODYNAMICS | 47 | 0.09 |
| DIAZEPAM + PREDNISOLONE | SECUNDARY | NOT AVAILABLE | PHARMACOKINETICS | 8 | 0.02 |
| DIAZEPAM + PREDNISONE | SECUNDARY | NOT AVAILABLE | PHARMACOKINETICS | 190 | 0.37 |
| DIAZEPAM + PROMETHAZINE | MODERATE | NOT AVAILABLE | PHARMACODYNAMICS | 605 | 1.18 |
| DIAZEPAM + PROPRANOLOL | SECUNDARY | NOT AVAILABLE | PHARMACOKINETICS | 412 | 0.80 |
| DIAZEPAM + QUETIAPINE | MODERATE | NOT AVAILABLE | PHARMACODYNAMICS | 1 | 0.00 |
| DIAZEPAM + RISPERIDONE | MODERATE | NOT AVAILABLE | PHARMACODYNAMICS | 667 | 1.30 |
| DIAZEPAM + RITONAVIR | MODERATE | REGULAR | PHARMACOKINETICS | 32 | 0.06 |
| DIAZEPAM + SERTRALINE | SECUNDARY | NOT AVAILABLE | PHARMACOKINETICS | 2542 | 4.97 |
| DIAZEPAM + TENOFOVIR + LAMIVUDINE + EFAVIRENZ | MODERATE | NOT AVAILABLE | PHARMACOKINETICS | 10 | 0.02 |
| DIAZEPAM + THIORIDAZINE | MODERATE | NOT AVAILABLE | PHARMACODYNAMICS | 51 | 0.10 |
| DIAZEPAM + TRAMADOL | IMPORTANT | REGULAR | PHARMACODYNAMICS | 173 | 0.34 |
| DIAZEPAM + VECURONIUM | MODERATE | NOT AVAILABLE | PHARMACODYNAMICS | 1 | 0.00 |
| DIAZEPAM + ZIDOVUDINE + LAMIVUDINE | SECUNDARY | NOT AVAILABLE | PHARMACOKINETICS | 11 | 0.02 |
| NITRAZEPAM + AMINOPHYLLINE | MODERATE | GOOD | PHARMACODYNAMICS | 1 | 0.00 |
| NITRAZEPAM + BROMOPRIDE | MODERATE | REGULAR | PHARMACOKINETICS | 2 | 0.00 |
| NITRAZEPAM + PHENOBARBITAL | MODERATE | GOOD | PHARMACODYNAMICS | 10 | 0.02 |
| NITRAZEPAM + METOCLOPRAMIDE | MODERATE | REGULAR | PHARMACODYNAMICS | 1 | 0.00 |
| NITRAZEPAM + MIDAZOLAM (Inj. Sol.) | MODERATE | REGULAR | PHARMACODYNAMICS | 2 | 0.00 |
| NITRAZEPAM + PERICIAZINE | IMPORTANT | REGULAR | PHARMACODYNAMICS | 6 | 0.01 |
| NITRAZEPAM + TRAMADOL | MODERATE | REGULAR | PHARMACODYNAMICS | 6 | 0.01 |
