# Supplementary material for: Consumption patterns and factors associated with inappropriate prescribing of benzodiazepines in Primary Health Care settings
Source: PLoS One. 2024 Sep 4;19(9):e0309984. doi: 10.1371/journal.pone.0309984 (PMC11373788; doi:10.1371/journal.pone.0309984)
Supplement: S2 Table — (DOCX) [file pone.0309984.s002.docx]

S2 Table. Detailed presentation of the dispensed drugs that interact with BZD, grouped according to their therapeutic classes (ATC).

| **ATC – First Level** | **ATC - Third Level** | **Drug** | **n** | **%** |
| --- | --- | --- | --- | --- |
| A - Drugs for the alimentary tract and metabolism | A02A - Antiacids | Aluminum hydroxide | 311 | 0.61 |
|  | A02B – Drugs for peptic ulcer and gastro-oesophageal reflux disease | Omeprazole | 5390 | 10.53 |
|  | A03F - Propulsives | Bromopride; Metoclopramide | 218 | 0.43 |
|  | A12A - Calcium | Calcium carbonate | 619 | 1.21 |
| C – Drugs for the cardiovascular system | C01A – Cardiac Glycosides | Digoxin | 70 | 0.14 |
|  | C01B – Antiarrhythmics, class I and III | Amiodarone | 227 | 0.44 |
|  | C01D - Vasodilators used in heart disease | Isosorbide dinitrate; Isosorbide mononitrate | 213 | 0.42 |
|  | C02A - Antiadrenergic agents, central action | Methyldopa; Clonidine | 756 | 1.48 |
|  | C02C - Antiadrenergic agents, peripheral action | Doxazosin | 222 | 0.43 |
|  | C02D - Agents that act on arteriolar smooth muscle | Hydralazin | 2 | 0.00 |
|  | C03A – Low-ceiling diuretics, Thiazides | Hydrochlorothiazide | 3094 | 6.04 |
|  | C03C - High-ceiling diuretics | Furosemide | 1125 | 2.20 |
|  | C03D - Aldosterone antagonists and other potassium-sparing agents | Spironolactone | 607 | 1.19 |
|  | C07A - Beta-blocking agents | Atenolol; Carvedilol; Esmolol; Metoprolol; Propranolol | 2411 | 4.71 |
|  | C08C - Selective calcium channel blockers, with mainly vascular effects | Amlodipine | 2200 | 4.30 |
|  | C09A - ACE inhibitors, plain | Captopril; Enalapril | 2196 | 4.29 |
|  | C09C - Angiotensin II receptor blockers, plain | Losartan | 3463 | 6.76 |
| G – Drugs for the genito urinary system and sex hormones | G03A - Hormonal contraceptives for systemic use | Estradiol + Norethisterone; Ethinylestradiol + Levonorgestrel; Norethisterone | 109 | 0.21 |
|  | G03C - Estrogens | Estradiol | 7 | 0.01 |
| H - Systemic hormonal preparations, excluding sex hormones and insulin | H02A - Corticosteroids for systemic use | Dexamethasone; Prednisolone; Prednisone | 201 | 0.39 |
| J - Anti-infective agents for systemic use | J01F - Macrolides, lincosamides and streptogramins | Clarithromycin; Erythromycin | 5 | 0.01 |
|  | J01M - Quinolone antibacterials | Ciprofloxacin | 100 | 0.20 |
|  | J02A - Antimycotics for systemic use | Fluconazole | 337 | 0.66 |
|  | J04A - Drugs for the treatment of tuberculosis | Isoniazid; Isoniazid + Rifampicin; Isoniazid + Rifampicin + Pyrazinamide + Ethambutol | 15 | 0.03 |
|  | J05A - Direct-acting antivirals | Darunavir; Efavirenz; Nevirapine; Ritonavir; Tenofovir + Lamivudine + Efavirenz; Zidovudine + Lamivudine | 163 | 0.32 |
| M – Drugs for the Musculo-skeletal System | M03A - Muscle relaxants, peripherally acting agents | Vecuronium | 2 | 0.00 |
|  | M03B - Muscle relaxants, centrally acting agents | Baclofen | 35 | 0.07 |
| N – Drugs for the Nervous System | N02A - Opioids | Tramadol | 346 | 0.68 |
|  | N02B - Other analgesics and antipyretics | Gabapentin | 1 | 0.00 |
|  | N03A - Antiepileptics | Valproic acid; Carbamazepine; Phenytoin; Phenobarbital | 3074 | 6.01 |
|  | N04A - Anticholinergic agents | Biperiden | 810 | 1.58 |
|  | N04B - Dopaminergic agents | Levodopa + carbidopa | 53 | 0.10 |
|  | N05A - Antipsychotics | Lithium carbonate; Chlorpromazine; Haloperidol; Levomepromazine; Periciazine; Quetiapine; Risperidone; Thioridazine | 5883 | 11.49 |
|  | N05C - Hypnotics and sedatives | Midazolam | 188 | 0.37 |
|  | N06A - Antidepressants | Amitriptyline; Imipramine; Bupropion; Clomipramine; Fluoxetine;  Nortriptyline; Sertraline | 15329 | 29.95 |
| R – Drugs for the Respiratory System | R03D - Other systemic drugs for obstructive airway diseases | Aminophylline | 48 | 0.09 |
|  | R06A - Antihistamines for systemic use | Dimenhydrinate + pyridoxine; Promethazine | 1358 | 2.65 |
| V - Various | V03A - All other therapeutic products | Flumazenil | 2 | 0.00 |
